# Specificity Protein 1-Mediated Promotion of CXCL12 Advances Endothelial Cell Metabolism and Proliferation in Pulmonary Hypertension

**DOI:** 10.3390/antiox12010071

**Published:** 2022-12-29

**Authors:** Evan R. DeVallance, Christopher M. Dustin, Daniel Simoes de Jesus, Imad Al Ghouleh, John C. Sembrat, Eugenia Cifuentes-Pagano, Patrick J. Pagano

**Affiliations:** 1 Department of Physiology and Pharmacology, West Virginia University School of Medicine, Morgantown, WV 26506, USA; 2 Center for Inhalation Toxicology, West Virginia University School of Medicine, Morgantown, WV 26506, USA; 3 Pittsburgh Heart, Lung, Blood and Vascular Medicine Institute, University of Pittsburgh, Pittsburgh, PA 15261, USA; 4 Department of Pharmacology and Chemical Biology, University of Pittsburgh, Pittsburgh, PA 15261, USA; 5 William Harvey Research Institute, Barts & The London School of Medicine and Dentistry, Queen Mary University of London, London EC1M 6BQ, UK; 6 Department of Cardiology, University of Pittsburgh, Pittsburgh, PA 15261, USA; 7 Division of Pulmonary, Allergy, and Critical Care Medicine, University of Pittsburgh, Pittsburgh, PA 15261, USA

**Keywords:** NOX1, CXCL12, Sp1, pulmonary arterial hypertension

## Abstract

Pulmonary arterial hypertension (PAH) is a rare yet devastating and incurable disease with few treatment options. The underlying mechanisms of PAH appear to involve substantial cellular proliferation and vascular remodeling, causing right ventricular overload and eventual heart failure. Recent evidence suggests a significant seminal role of the pulmonary endothelium in the initiation and promotion of PAH. Our previous work identified elevated reactive oxygen species (ROS)-producing enzyme NADPH oxidase 1 (NOX1) in human pulmonary artery endothelial cells (HPAECs) of PAH patients promoting endothelial cell proliferation in vitro. In this study, we interrogated chemokine CXCL12′s (aka SDF-1) role in EC proliferation under the control of NOX1 and specificity protein 1 (Sp1). We report here that NOX1 can drive hypoxia-induced endothelial CXCL12 expression via the transcription factor Sp1 leading to HPAEC proliferation and migration. Indeed, NOX1 drove hypoxia-induced Sp1 activation, along with an increased capacity of Sp1 to bind cognate promoter regions in the CXCL12 promoter. Sp1 activation induced elevated expression of CXCL12 in hypoxic HPAECs, supporting downstream induction of expression at the CXCL12 promoter via NOX1 activity. Pathological levels of CXCL12 mimicking those reported in human PAH patient serum restored EC proliferation impeded by specific NOX1 inhibitor. The translational relevance of our findings is highlighted by elevated NOX1 activity, Sp1 activation, and CXCL12 expression in explanted lung samples from PAH patients compared to non-PAH controls. Analysis of phosphofructokinase, glucose-6-phosphate dehydrogenase, and glutaminase activity revealed that CXCL12 induces glutamine and glucose metabolism, which are foundational to EC cell proliferation. Indeed, in explanted human PAH lungs, demonstrably higher glutaminase activity was detected compared to healthy controls. Finally, infusion of recombinant CXCL12 into healthy mice amplified pulmonary arterial pressure, right ventricle remodeling, and elevated glucose and glutamine metabolism. Together these data suggest a central role for a novel NOX1-Sp1-CXCL12 pathway in mediating PAH phenotype in the lung endothelium.

## 1. Introduction

Pulmonary arterial hypertension (PAH) is a rare chronic and progressive disease of the pulmonary vasculature that affects a relatively young population and is particularly deadly with a 5-year survival rate at approximately 60%, similar to that for stroke and notably worse than that for breast and colon cancer [1,2]. The annual cost per patient in advanced stages can exceed $200 K/year (USD) [3,4]. Despite strides made in PAH research and FDA-approval of drugs palliative to the condition, i.e., vasodilators [5], there is still no cure. This lack of a cure for PAH predictably arises out of the complexity of cellular processes in the disease that converge to cause hyperproliferative remodeling of the pulmonary vasculature. Excessive inward vascular remodeling and obstructive lesions increase pulmonary vascular resistance and pulmonary artery pressure which, in turn, causes right ventricular overload and ultimately death due to right ventricular failure. Thus, a search for signaling mediators whose targeting disrupts pulmonary vascular remodeling and cures rather than palliates PAH remains elusive.

Recent work has identified a seminal role for pulmonary endothelial cells (ECs) in the initiation and progression of PAH [6,7,8,9]. Among the factors involved, reactive oxygen species (ROS) have emerged as central mediators of this progression [10,11,12]. ROS are robustly generated by NADPH Oxidases (NOX, including NOX1-5, DUOX1, and DUOX2): multicomponent transmembrane enzyme complexes that expressly generate superoxide (O_2_^−^) rapidly dismuted to hydrogen peroxide (H_2_O_2_) [13]. H_2_O_2_ and other ROS trigger an array of effector molecules [14], whose excitation initiates deleterious effects causative to disease. In PAH, NOX has been shown by our group and others to drive hyperproliferation and migration [15]. Indeed, our studies previously linked NOX1 to endothelial cell proliferation and migration in PAH [8,15]. However, the signaling pathways downstream of NOX1 remain largely unexplored.

Recently, analyses of human plasma revealed a strong correlation between increased expression of the chemokine CXCL12 (also known as SDF-1) with PAH severity [16,17,18]. Notably, CXCL12 is implicated in tumor cell proliferation and pro-proliferative metabolic changes [19,20,21]. Intriguingly, remarkably common parallels have been drawn between cancer and PAH [22]. In contrast to what has been defined in cancer, the signaling pathways regulating CXCL12 and EC proliferation in PAH remain unclear. 

Studies in non-vascular cell types provided us clues that CXCL12 expression in the vascular endothelium could plausibly be controlled by a putative redox-sensitive transcription factor specificity protein 1 (Sp1) [23,24,25,26,27]. This raised the potential for CXCL12 to be controlled by NOX1 and Sp1 in lung ECs. In the current study, our novel findings imply that hypoxia-induced NOX1 triggers Sp1 and CXCL12. Furthermore, the data implicate Sp1 and CXCL12 under the control of NOX1 in EC-centered remodeling and metabolic perturbations emblematic of PAH in human cells, live mice and explanted human lungs.

## 2. Methods

### 2.1. Reagents

Bovine liver catalase was purchased from Sigma–Aldrich (St. Louis, MO, USA.). Protease and phosphatase inhibitor cocktail tablets were purchased from Roche Diagnostics GmbH (Mannheim, Germany). Silencer-select siRNAs for NOX1 (s25728) and CXCL12 (n287425) were purchased from ThermoFisher (Waltham, MA, USA). Validated Stealth siRNA for Sp1 (VHS40865) was purchased from ThermoFisher (Waltham, MA, USA). Antibodies against p-Sp1 (phospho-T453) (ab59257), Sp1 (ab13370), CXCL12 (ab9797), CXCL12 (Alexa Fluor^®^ 488) (ab217985) antibodies were purchased from Abcam (Cambridge, MA, USA). β-actin (sc-47778) antibodies were purchased from Santa Cruz Biotechnology, Inc. (Dallas, TX, USA.). p-mTOR S2448 (2971S) and mTOR (2972) antibodies were purchased from Cell Signaling Technology (Danvers, MA, USA). Goat anti-rabbit (925–68070), and goat anti-mouse (925–68071) secondary antibodies were purchased from LI-COR Biosciences (Lincoln, NE, USA). Assessment of CXCL12 by ELISA was conducted with a Quantikine^®^ ELISA (DSA00) kit from R&D systems Inc. (Minneapolis, MN, USA). GLS1 inhibitor C-839 was purchased from EMD Millipore Corp (Burlington, MA, USA). Mithramycin A (11434) was purchased from Cayman Chemical (Ann Arbor, MI, USA). TransAM^®^ Transcription Factor Activation Assay for Sp1 was purchased from Active Motif (Carlsbad, CA, USA). Enzymatic activity kits were purchased from Sigma–Aldrich (St. Louis, MO, USA.) for G6PD (MAK015), Cohesion Bioscience (London, UK) for GLS1 (CAK1065) and Abcam (Cambridge, MA, USA) for PFK (ab155898). Glucose uptake kit (ab235976) was purchased from Abcam (Cambridge, MA, USA) and Click-iT™ EdU Cell Proliferation Kit (C10420) was purchased from Invitrogen™ ThermoFisher (Waltham, MA, USA). BD Cytofix/Cytoperm™ Fixation/permeabilization Solution Kit was purchased from Fisher Scientific (Waltham, MA, USA). Recombinant CXCL12 was purchased from Abcam (human ab9798; Cambridge, MA, USA.) and R&D systems Inc. (mouse 460-SD) (Minneapolis, MN, USA). CBA (Coumarin 7-Boronic Acid; 1357078–03-5) was purchased from Cayman Chemical (Ann Arbor, MI, USA). Dehydroepiandrosterone (DHEA, D-063) was purchased from Sigma.

### 2.2. Human Samples

Lung sections from subjects undergoing lung transplant were generously provided by Dr. Elena Goncharova in association with the University of Pittsburgh’s Pulmonary Arterial Hypertension Bio-Bank under protocols approved by the University of Pittsburgh Institutional Review Board. Samples were de-identified and only demographic information made available. Idiopathic PAH (PAH) vs. non-PAH human subject lung samples were age and sex matched (Table S1. Controls: n = 6–10, Age- 52 +/− 14, 54% female and PAH: n = 6–10, Age- 56 +/− 13, 60% female). Tissues were weighed and processed in either complete RIPA (containing 0.1 mM protease and phosphatase inhibitors, Roche) for Western blot analysis or in PBS containing 0.1 mM protease and phosphatase inhibitors for enzymatic activity assays or ELISA.

### 2.3. Cell Culture

Three separate lots (701035, 708987, and 657513) of human pulmonary artery endothelial cells (hPAECs- Lonza, Walkersville, MD, USA) were grown between passages 3 and 6 in complete EBM media containing EGM bullet kit and serum-synchronized in 1:10 serum reduced EBM media (Lonza Walkersville, MD, USA) overnight or for 4 h. Full medium was restored to cells which were then incubated in a normoxic (21% O_2_) CO_2_ cell incubator or in a hypoxia chamber (1% O_2_) for 24 or 48 h. For gene manipulation, hPAECs grown in 6-well plates at 70–80% confluence were subjected to 10 nM siRNA against NOX1, Sp1, CXCL12, or scrambled control delivered by lipofectamine 3000 transfection reagent (Life Technology) according to manufacturer’s protocol. Following experimental treatments, cells for Western blot analysis were immediately lysed and collected on ice in complete RIPA buffer. For enzymatic activity assays and ELISAs, cells were collected into PBS containing 0.1 mM protease and phosphatase inhibitors. For flow cytometry, cells were washed and fixed as described below.

### 2.4. In Vivo CXCL12 Infusion, mPAP and RV Hypertrophy 

All animal procedures were approved by the University of Pittsburgh’s IACUC (Protocol #20077336). Female C57BL/6J mice at 8–9 weeks of age were randomly assigned to either saline control or mouse recombinant CXCL12 (mrCXCL12) groups. Model 1004 minipumps from Alzet were loaded with 0.07 mg/mL mrCXCL12 infused at a rate of 7.7 ng/h; controls were delivered physiological saline. Prior to implantation, all pumps were primed in physiological saline 24 h. Mice were anesthetized with isoflurane and a subscapular incision was made for subcutaneous minipump insertion. The incision was then sutured, and mice were monitored for recovery. After 3 wks of treatment, hemodynamic measurements were collected via intracardiac insertion of a Transonic admittance pressure–volume catheter. Terminal procedures were conducted on fully anesthetized mice whose extent was verified by toe pinch. Mice were placed on a heating pad and a tracheal tube was inserted for ventilation and isoflurane delivery. Next, a thoracotomy was performed and the rib cage was retracted to expose the heart. The transonic catheter was then advanced into the right ventricle (RV) to measure right ventricle systolic pressure (RVSP), which was used to calculate mean pulmonary arterial pressure (mPAP). Immediately following the acquisition of mPAP, the heart was excised, and the RV was dissected away from the left ventricle (LV) and septum (S). Heart tissues were weighed to obtain wet weight and calculate Fulton index (RV/LV+S). Additionally, the lungs were removed and whole lung lysates were prepared in PBS containing 0.1 mM protease and phosphatase inhibitors to assess enzymatic activity. All measurements were collected in a blinded fashion.

### 2.5. Protein Expression (Western Blot, ELISA, and Flow Cytometry)

Western blotting was performed as previously described [8]. Briefly, complete RIPA buffer was added to plated hPAECs following removal of medium and cells were scraped from plates. 30 μg of total protein from lysates was combined with 5X Laemmli sample buffer (Bio-Rad, Hercules, CA, USA) containing β-mercaptoethanol and boiled at 95 °C for 5 min. Samples were then resolved by SDS-PAGE and transferred to 0.2 μm nitrocellulose membrane. Membranes were blocked with LiCOR Odyssey blocking buffer and probed with primary antibodies against: CXCL12, p-Sp1, Sp1, p-mTOR, mTOR, β-actin. Membranes were probed with LiCOR near-infrared secondary antibodies and imaged using LICOR Odyssey. Optical density was assessed by ImageJ software, normalized against β-actin control, and represented as fold change from control group. 

For ELISA analyses, appropriate lysates of either human tissue or hPAECs were analyzed utilizing the R&D systems Quantikine ELISA (DSA00, R&D Systems) for CXCL12 according to manufacturer instructions. Absorbance was read on a Synergy 4 Plate reader at 450 nm.

For flow cytometry, a single cell suspension was prepared by trypsinizing cells and resuspending into PBS. Cells were pelleted by centrifugation, washed twice with PBS, and vortexed to fully dissociate the pellet in 100 μL of BD Cytofix/Cytoperm solution (BD Biosciences). Cells were incubated for 20 min prior to washing twice with buffer containing BD Perm/Wash buffer (BD Biosciences), followed by addition of 1 mL of 1X permeabilization buffer. Cells were centrifuged at 400–600× *g* for 5 min at room temperature and supernatant was discarded. The permeabilization step was repeated once. Cells were then resuspended in BD Perm/Wash buffer containing a 1:500 dilution Alexa Fluor^®^ 488 anti-SDF1 (CXCL12) antibody (ab217985) or appropriate negative control and incubated at 4 °C for 30 min in the dark. Cells were washed twice with BD Perm/WashTM buffer, away from light, before being resuspended in eBioscience Flow Cytometry Staining Buffer (Invitrogen). An equal number of cells (10,000) were assayed per treatment group. A shift to higher mean FITC-A (shift in fluorescence) indicates higher quantities of CXCL12. Antigen expression (mean fluorescence) was detected utilizing BD LSRFortessa flow cytometer and each sample was then normalized to control. Areas under the curve remain the same while mean fluorescence changes are depicted by shifts and their quantification.

### 2.6. ROS Measurement

Coumarin boronate assay (CBA) was performed on explanted lung tissue using a protocol adapted and modified from Zielonka and coworkers [28,29] and as previously reported [8,30,31,32]. Lung tissue was homogenized using a glass-on-glass mortar and pestle followed by 5 rounds of freeze thaw and trituration (30-gauge needle). 1–2 μg of protein was loaded into a 384-well, black-sided clear bottom plate with assay buffer consisting of HBSS with 25 mM HEPES, 1% BSA, 10 μM EDTA, 100 μM L-NAME and 1 mM taurine with and without the NOX1 inhibitor NOXA1ds at 10 μM. Finally, 5X CBA probe solution with and without 1 KU catalase were added to the wells and the plate was placed in a Biotek plate reader preheated to 37 °C and read kinetically (each minute for 3 h) at λex = 350 nm and λem = 450 nm. The average rate of fluorescence in its linear phase was determined and background from catalase control samples was subtracted. 

### 2.7. TransAM Assay

To measure the activity of the transcription factor Sp1 binding to DNA, we utilized the TransAM assay from ActiveMotif, which contains synthetic DNA repeats of consensus binding sites for Sp1. Lysates prepared from explanted lung samples or hypoxic hPAECs (with or without siRNA against NOX1) were added to the plate and processed according to the manufacturer’s protocol. This allows for the determination of an activated Sp1 that would be capable of binding and activating a promoter in a sample, i.e., its binding to promoter consensus sequence-coated wells per TransAM. It is not an indicator of *in cellulo* promoter binding. Data was acquired by absorbance measured at 450 nm in a Biotek Synergy 4 plate reader. Data were normalized to normoxic control in hPAEC experiments and to healthy sex/age matched controls for explanted lung samples. 

### 2.8. Cell Proliferation

Cell proliferation was measured by both crystal violet (CV) and Click-iT^TM^ EdU assays. For measurement of proliferation via CV, as previously reported [8], cells were washed with PBS and incubated in CV solution for 20 min following treatment, washed with PBS, and then dried. Finally, CV was then solubilized in methanol and absorbance read at 595 nm. Data were expressed as fold change from control. For EdU measurements, hPAECs were pre-treated with 2 μM EdU according to the manufacturer’s protocol and immediately treated with hypoxia or human recombinant CXCL12 (hrCXCL12) in full medium for 24 h. Cells were trypsinized and collected in 1.5 mL Eppendorf tubes. Cells were fixed and stained using the Invitrogen Click-iT EdU Cell Proliferation Kit according to the manufacturer’s protocol. EdU-positive cells were detected utilizing BD LSRFortessa flow cytometer and per cent of positive cells was calculated for each sample with treatment group means plotted, with proliferating cells indicated by a significant rightward shift along the *x* axis of resultant intensity plots (Figure S1F).

### 2.9. Luciferase Reporter Assay

Due to the intractability of transfection of hPAECs, human umbilical vein endothelial cells (HUVEC) in 96-well plates were transfected with LightSwitch reporter constructs containing the Sp1 synthetic response element or the CXCL12 promoter reporter construct (SwitchGear Genomics; Carlsbad, CA, USA) chimerized with the luciferase gene for proof-of-concept promoter binding studies. Readings were taken according to the manufacturer’s protocol, in cells treated with or without the addition of NOX1- or Sp1-targeted siRNA for 24 h. Cells were subsequently incubated under normoxic or hypoxic conditions for 24 h. Medium was removed and cells were washed once with PBS prior to freezing at −80°C for improved cell lysis. Cells were then brought to RT and incubated with LightSwitch assay buffer containing luciferase substrate for 30 min. Luminescence was measured using a Biotek Synergy 4 plate reader.

### 2.10. Migration; Wound Healing Assay

Wound healing assays were performed as previously reported [8]. In brief hPAECs in 6-well plates were serum-deprived for 4 h followed by creation of a gap/scratch in the EC monolayer using a P1000 pipette tip followed by replacement of complete media. Plates were placed in a cell culture incubator under normoxic conditions or into a hypoxia chamber in the incubator for 24 h. The scratch was imaged at time point 0 and at 24 h. Each well was imaged at 3 distinct fields of view across all samples and at both time points. The percent closure was calculated at each coordinate and then averaged together for the total well’s percent closure. Data are presented as percent closure of the wound area (100 × [area_t0_−area_t24_]/area_t0_) comparing treatment vs. control at 24 h. 

### 2.11. Metabolic Enzyme Activity

The enzymatic activity of cell and tissue lysates were tested using the G6PD activity kit (Sigma), PFK activity kit (Abcam), and GLS1 activity kit (Cohesion Biosciences). Lysates were prepared and loaded into 96-well plates and manufacturer’s protocols were followed for all kits. Activity was quantified by kinetic absorbance readings in a BioTek Synergy 4 plate reader. Activity was calculated from the standard curve of each respective assay kit and displayed as μmol/min (PFK), ng/mL/min (G6PD), and U/mg (GLS1).

### 2.12. Glucose Uptake

hPAECs were incubated in glucose free media for 4 h (also containing treatment). After 4 h, media was spiked with 1 mM fluorescently labeled glucose analog of 2-(*N*-(7-Nitrobenz-2-oxa-1,3-diazol-4-yl) Amino)-2-deoxyglucose (2-NBDG) from the Abcam glucose uptake kit. Cells were incubated with the 2-NBDG for 10 min, washed with PBS, and prepared for flow cytometry following the manufacturer’s protocol. Mean fluorescent intensity was measured for 10,000 cells in each treatment group by BD LSRFortessa flow cytometer.

### 2.13. Statistical Analysis

Results are reported as mean ± S.E.M. and analyzed using GraphPad Prism software (v8). Analysis of hypoxia, siRNA treatment, and drug treatment experiments were conducted by one-way ANOVA with Holm–Sidak post hoc analysis to determine differences between groups. Comparison between 2 groups was analyzed by unpaired Student t-test. Finally, the impact of NOXA1ds treatment on ROS production in lung lysates was analyzed by repeated measures ANOVA. A *p* value of *p* = 0.05 or lower was taken as indicative of statistical significance.

## 3. Results

### 3.1. NOX1 Induces CXCL12 in Hypoxic hPAECs and Human PAH

**Figure 1:**
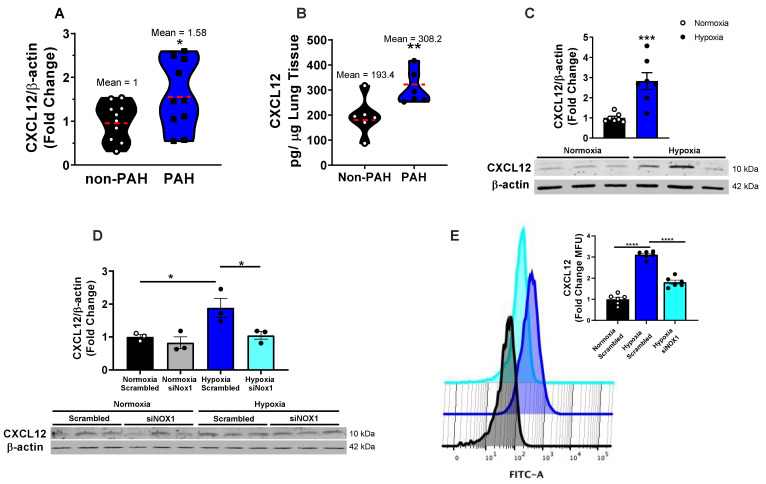
**CXCL12 expression is elevated in PAH and is NOX1-dependent in hypoxic pulmonary endothelial cells.** (**A**) Western blot analysis of CXCL12 expression in explanted lung tissue samples from PAH or non-PAH patients (n = 6–10). (**B**) ELISA analysis of CXCL12 levels in explanted human lung tissue samples from PAH and non-PAH patients (n = 6–10). Mean values are indicated by a dotted red line. (**C**) Cultured human pulmonary artery endothelial cells (hPAECs) were exposed to 1% O_2_ (hypoxia) or normoxia for 48 h and CXCL12 expression was assessed by Western blot (n = 7). D-E. hPAECs were transfected with scrambled or NOX1-targeted siRNAs for 16 h. Cells were exposed to 1% O_2_ or normoxia and CXCL12 expression was assessed by (**D**). Western blot or (**E**). flow cytometry (n = 3–6). Representative flow cytometry histograms depict mean fluorescent intensity of FITC along the *x* axis, with a rightward shift of the peak indicating increased CXCL12 expression. Results analyzed by one-tailed t-test (**A**–**C**), and one-way ANOVA followed by Holm–Sidak post hoc analysis to determine differences between treatment groups (**D**,**E**) * *p* < 0.05, ** *p* < 0.01, *** *p* < 0.001, **** *p* < 0.0001.

CXCL12 levels in PAH lung samples were elevated ca. 1.5-fold as measured by both Western blot and ELISA, compared to sex and age matched non-PAH controls (Figure 1A,B and Figure S1A), consistent with previous findings [16,18]. We previously reported that NOX1 is markedly elevated in PAH [15]. To recapitulate PAH phenotype in vitro, hPAECs were subjected to hypoxia vs. normoxia. CXCL12 expression was significantly elevated at 48 h of hypoxia (*p* < 0.001) and validated with siCXCL12 siRNA (Figure 1C–E); an approximate 3- & 2-fold increase, respectively (Figure 1C,D). NOX1 siRNA-treatment for 16 h prior to hypoxia abolished elevated CXCL12 expression measured by Western blot and flow cytometry (Figure 1D,E, respectively); NOX1 siRNA had no effect on basal CXCL12 under normoxic conditions (Figure 1D) but markedly attenuated the hypoxia-induced elevation in CXCL12 (Figure 1D,E) With respect to ROS, our previous data from PAH vs. non-PAH human subjects demonstrated increased ROS levels in PAH that were NOX1-mediated [8,15]. Indeed, lysates of explanted lungs from human subjects in the current study corroborated those data (Figure S1B).

### 3.2. NOX1 Mediates SP1-Induced CXCL12 in PAH-Associated Endothelial Signaling

**Figure 2:**
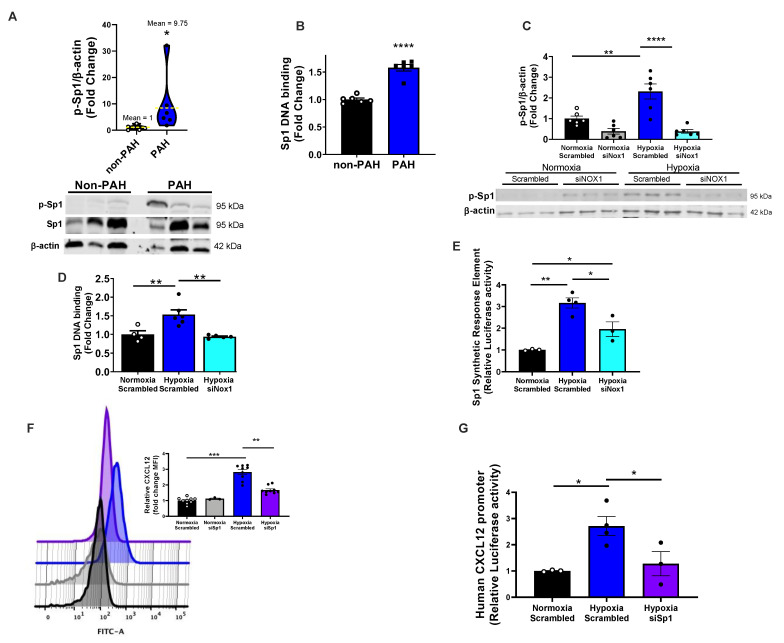
**NOX1 activates the transcription factor specificity protein 1 (Sp1), Sp1 consensus DNA binding, and CXCL12 gene promotion.** (**A**) Western blot analysis of human lung lysates for p-Sp1/β-actin (n = 6). (**B**) Sp1 consensus DNA binding activity in human lung lysates measured by Active Motif TransAM assay (n = 6). (**C**) hPAECs transfected with scrambled or NOX1-targeted siRNA for 24 h were exposed to 1% O_2_ and analyzed by Western blot for Sp1 activation, p-Sp1/β-actin (n = 3–6). (**D**) hPAECs transfected with scrambled or NOX1-targeted siRNA for 24 h were exposed to 1% O_2_ and analyzed by Active Motif Trans AM assay. (**E**) Sp1 transcriptional activation was tested in HUVECs transfected with SpRE luciferase plasmid in addition to scrambled or NOX1-targeted siRNA. Cells were placed in normoxia or 1% O_2_ for 48 h and luciferase activity was measured by plate reader luminometer (n = 3–6). (**F**) hPAECs transfected with scrambled or Sp1-targeted siRNA for 24 h were exposed to 1% O_2_ 48 h. CXCL12 expression was measured by flow cytometry (n = 3–9). Representative flow cytometry histograms depict mean fluorescent intensity of FITC along the *x* axis, with a rightward shift of the peak indicating increased CXCL12 expression. (**G**) HUVECs transfected with luciferase reporter construct containing 1 kb of the human CXCL12 promoter were transfected with scrambled or Sp1-targeted siRNA. Cells were then exposed to 48 h hypoxia (1% O2) and Sp1′s ability to promote luciferase activity measured by plate reader luminometer (n = 3–4). Results were analyzed by Student t-test (**A**,**B**), one-way ANOVA followed by the Holm–Sidak test (**C**–**G**) * *p* < 0.05, ** *p* < 0.01, *** *p* < 0.001, **** *p* < 0.0001.

Previous reports in other tissues suggested Sp1 is a redox-sensitive transcription factor in cortical neurons [27], drosophila epithelia and human gingival fibroblasts [33]. Other studies inferred that Sp1 may drive CXCL12 expression in human astrocytoma cells, human lung fibroblasts, and rat β-cells [23,24]. Thus, we interrogated a role for Sp1 as a potential link between NOX1 and CXCL12. Although there was no significant change in total expression of Sp1 in PAH vs. non-PAH controls (Figure S1D) a significant (*p* < 0.05) increase in Sp1 phosphorylation at T453 was observed indicative of its activation (Figure 2A and Figure S1D). PAH samples were analyzed by TransAM assay to test the binding of Sp1 to a consensus Sp1 DNA response element (SpRE). Consistent with the observed increase in pT453 Sp1, PAH patient lung samples exhibited elevated binding of Sp1 to SpRE (Figure 2B). Hypoxia also induced a roughly 2-fold increase in p-T453 Sp1 (phosphorylation/activation) compared to controls in vitro, while siNOX1 abrogated this increase (Figure 2C), consistent with NOX1 driving Sp1 activation. 

Hypoxia-induced binding of Sp1 to SpRE in the TransAM assay was prevented by NOX1 silencing (Figure 2D). Similarly, SpRE luciferase constructs transfected into HUVEC showed greater activation in hypoxic ECs, which was halved by NOX1 silencing (Figure 2E). To interrogate a role for Sp1 as a causal link between NOX1 and CXCL12, hPAECs transfected with either scrambled or Sp1 siRNA were exposed to hypoxia. Sp1 silencing caused a greater than 90% knockdown of Sp1 (Figure S1E) and disrupted CXCL12 expression, measured by flow cytometry (Figure 2F). Further, using hPAECs transfected with luciferase reporter plasmids containing the 5′ region of the human CXCL12 promoter, cells exposed to hypoxia demonstrated a robust increase in promoter activity that was brought to baseline by Sp1 knockdown (Figure 2G). Overall, these results suggest that Sp1 is a hypoxia-activated transcription factor that regulates endothelial CXCL12 expression downstream of NOX1.

### 3.3. CXCL12 Promotes hPAEC Proliferation/Migration

Human recombinant CXCL12 (hrCXCL12) induced a concentration-dependent increase in hPAEC proliferation measured by crystal violet assay (Figure S2A). Supporting clinical relevance of our findings, subsequent experiments were conducted using 3 ng/mL CXCL12, a concentration that is consistent with CXCL12 levels observed in PAH patient plasma [16,17]. Twenty four-hour exposure to this pathological concentration of hrCXCL12 robustly increased hPAEC proliferation (Figure S2B,C) compared to vehicle control. Scratch wound closure, an assay used to predominantly assess EC migration in response to hrCXCL12, progressed at a greater than 2-fold faster rate than controls (Figure S2D). 

**Figure 3:**
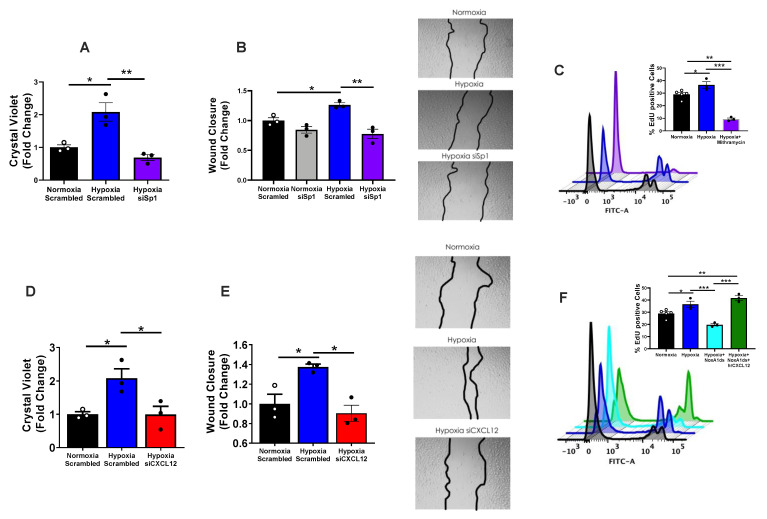
**Hypoxia-induced proliferation and migration of pulmonary endothelial cells are NOX1-, Sp1- and CXCL12-dependent.** (**A**,**B**) Normoxic vs. hypoxic hPAECs (1% O_2_) were transfected with either scrambled or Sp1-targeted siRNA and analyzed by (**A**) Crystal Violet and (**B**). Wound closure assay; insets are wound images taken at 24 h. (**C**) hPAECs were subjected to normoxic vs. hypoxic (1% O_2_) conditions following treatment with vehicle (DMSO) or Sp1 inhibitor mithramycin; proliferation was analyzed by Click-It^TM^EdU incorporation and flow cytometry. (**D**,**E**) hPAECs transfected with scrambled or CXCL12-targeted siRNA analyzed by (**D**). Crystal Violet and (**E**). wound closure assay. (**F**) hPAECs were treated with scrambled or Nox1-selective inhibitory peptide NoxA1ds, in addition to recombinant CXCL12 or vehicle control (PBS),and were subjected to hypoxia (1% O_2_) or normoxia. Proliferation was analyzed by Click-It EdU incorporation and flow cytometry. Representative flow cytometry histograms depict populations of EdU-positive and -negative cells in a sample along the *x* axis, with right hand peaks indicating proliferating cells taken as percentage to the total number of cells in the sample (see Figure S1F for gating). Results were analyzed by one-way ANOVA and Holm–Sidak post hoc analysis (**A**–**F**). * *p* < 0.05, ** *p* < 0.01, *** *p* < 0.001.

Elevated proliferation and wound closure in hypoxic hPAECs were neutralized by siSp1 (Figure 3A,B). Interestingly, Sp1 DNA binding antagonist mithramycin A (Cayman Chemical) corroborated these results by virtually eradicating (below baseline) EdU-positive hPAECs post-hypoxia (Figure 3C and Figure S1F for gating). Moreover, targeting of Sp1 downstream mediator, CXCL12, by siRNA markedly reversed hypoxia-elevated proliferation and wound closure (Figure 3D,E). To more thoroughly test whether NOX1-augmented proliferation involves CXCL12, we examined whether hrCXCL12 might restore a suppressed hypoxia-mediated proliferation in hPAECs following NOX1 inhibition. Firstly, consistent with findings revealed with NOX1 siRNA in Figure 2, selective NOX1 inhibitor NoxA1ds halved hypoxia-induced endothelial proliferation (Figure 3F). Importantly, treatment with 3 ng/mL hrCXCL12 following NOXA1ds administration restored hypoxia-induced proliferation. Taken together, these data support that autocrine/paracrine CXCL12 signaling is central to lung endothelial cell proliferation, and that NOX1 and Sp1 control this signaling.

### 3.4. CXCL12 Activates Pro-Proliferative Metabolic Pathways

**Figure 4:**
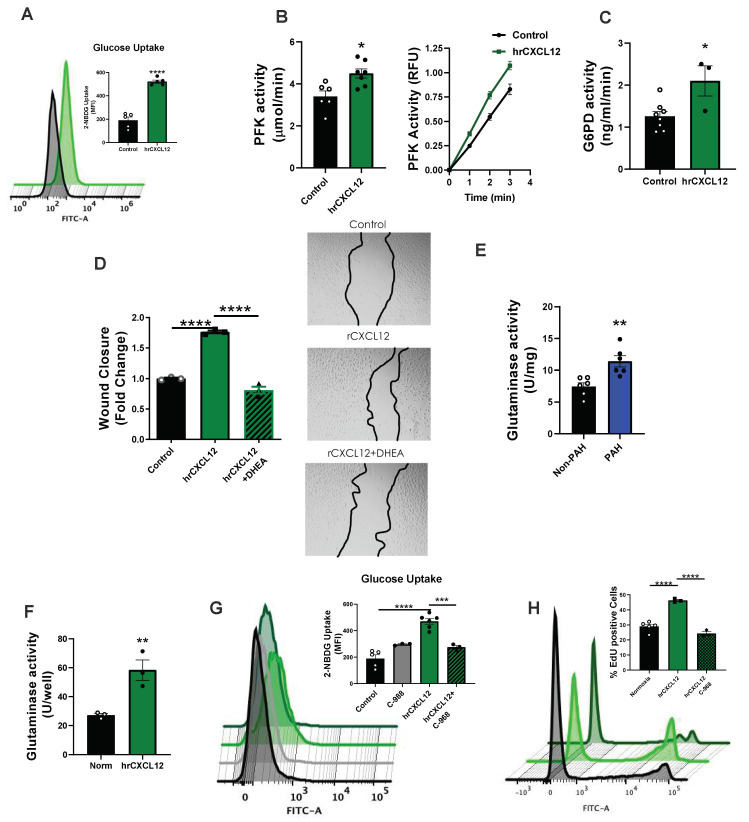
**CXCL12 induces pro-proliferative metabolic activity**. (**A**) hPAECs were treated with recombinant CXCL12 for 24 h and glucose uptake was analyzed by fluorescent NBGD using flow cytometry. Representative flow cytometry histograms depict mean fluorescent intensity of FITC along the *x* axis, with a rightward shift of the peak indicating increased glucose uptake. (**B**) Phosphofructokinase (with RFU/min kinetic activity, inset) and (**C**) glucose-6-phosphate dehydrogenase (G6PD) activity were tested in hPAECs treated with hrCXCL12 vs. control. (**D**) Wound closure was measured in hPAECs treated with hrCXCL12 along with the glucose-6-phosphate dehydrogenase inhibitor DHEA or vehicle (methanol); inset shows images at 24 h. (**E**) Lung lysates from age- and sex-matched PAH and non-PAH subjects were assessed for glutaminase activity (n = 6). (**F**) hPAECs treated with hrCXCL12 were assessed for glutaminase activity after 24 h stimulation. (**G**,**H**) hPAECs treated with hrCXCL12 plus or minus the glutaminase inhibitor C-968 or vehicle (DMSO) to measure (**G**). glucose uptake (fluorescent NBGD-flow cytometry) and (**H**). proliferation (Click-it Edu-flow cytometry). Representative flow cytometry histograms in **H** depict populations of EdU-positive and -negative cells in a sample along the *x* axis, with a righthand peak cluster indicating proliferating cells taken as percentage to the total number of cells in the sample (for gating see Figure S1F). Results were analyzed with Student t-test (**A**–**C**,**E**,**F**) and one-way ANOVA and Holm–Sidak post hoc analysis (**D**,**G**,**H**) * *p* < 0.05, ** *p* < 0.01, *** *p* < 0.001, **** *p* < 0.0001.

Numerous reports implicate glycolytic upregulation (Warburg effect) in PAH fueling a propensity for proliferation (reviewed in [4,34]). We thus explored the contribution of CXCL12 to these pathways. Treatment of normoxic hPAECs with 3 ng/mL CXCL12 for 24 h significantly (*p* < 0.0001) increased the uptake of fluorescence-labeled glucose analog 2-NBDG (Figure 4A). Moreover, we quantified the activity of phosphofructokinase (PFK), the rate limiting enzyme in glycolysis, and glucose 6-phosphate dehydrogenase (G6PD), as it is rate limiting for the pentose phosphate pathway, two pathways germane to pro- pro-proliferative signaling [34]. hPAECs treated with CXCL12 displayed elevated rates of PFK and G6PD activity when compared to vehicle-treated controls (Figure 4B,C). G6PD has previously been shown to cycle back into glycolysis and generates an essential cofactor (NADPH) in multiple enzyme systems involved in angiogenic EC proliferation [35]. We therefore performed a wound closure assay to determine if hrCXCL12-induced G6PD activity is essential to this process. Indeed, hPAECs treated with G6PD inhibitor dehydroepiandrosterone (DHEA) reverted hrCXCL12-mediated wound closure to below baseline (Figure 4D). 

The role of pro-proliferative glutamine metabolism in PAH has been less-well studied. Activity of glutaminase (the rate-limiting enzyme in the glutamine pathway) was found to be elevated in human lung samples from PAH patients compared to controls (Figure 4E). Treatment of hPAECs with hrCXCL12 for 24 h similarly increased the glutaminase activity over 2-fold when compared to vehicle control (Figure 4F). In light of recent evidence supporting crosstalk between glutamine and glucose metabolism [36,37], we tested whether glutaminase activity impinged on hPAEC glucose uptake. hPAECs were pre-treated with the glutaminase inhibitor C-968 [38] or vehicle control for 20 min followed by hrCXCL12 for 24 h. Glucose uptake stimulated by hrCXCL12 treatment was largely abolished by C-968 (Figure 4G). Phenotypically, hrCXCL12-stimulated proliferation was completely prevented by treatment with C-968 (Figure 4H). C-968 effected an insignificant change in basal glucose uptake. Taken together, these data indicate that CXCL12 directly feeds into pro-proliferative metabolic alterations relevant for PAH.

### 3.5. CXCL12 Activates the Master Metabolic Regulator mTOR in hPAECs

Upon detecting the activation of multiple pro-proliferative metabolic pathways following CXCL12 administration, we questioned whether CXCL12 could activate mTOR and whether mTOR is involved in CXCL12-mediated EC proliferation and migration. Indeed, mTOR is implicated in increased glycolysis and glutamine metabolism proliferation cancer cells [39,40,41]. Treatment of hPAECs with hrCXCL12 induced robust phosphorylation of mTOR at serine-2448 compared to vehicle control, indicative of increased mTOR activity [42,43] (Figure S3A). Next, we treated hPAECs with the mTOR inhibitor rapamycin and assessed hrCXCL12-induced proliferation and wound closure. Rapamycin significantly (*p* < 0.0001) reduced the number of EdU-positive cells induced by hrCXCL12 (Figure S3B). Furthermore, rapamycin treatment significantly (*p* < 0.01) blunted hrCXCL12-induced wound closure (Figure S3C) compared to untreated cells. These results suggest that CXCL12 signaling involves downstream activation of mTOR, which is involved in endothelial proliferation and migration. 

### 3.6. Infusion of rCXCL12 in Mice Causes an Increase in Pulmonary Artery Pressure, Cardiac Remodeling and Increased Glutaminase and PFK Activity in the Lung

**Figure 5:**
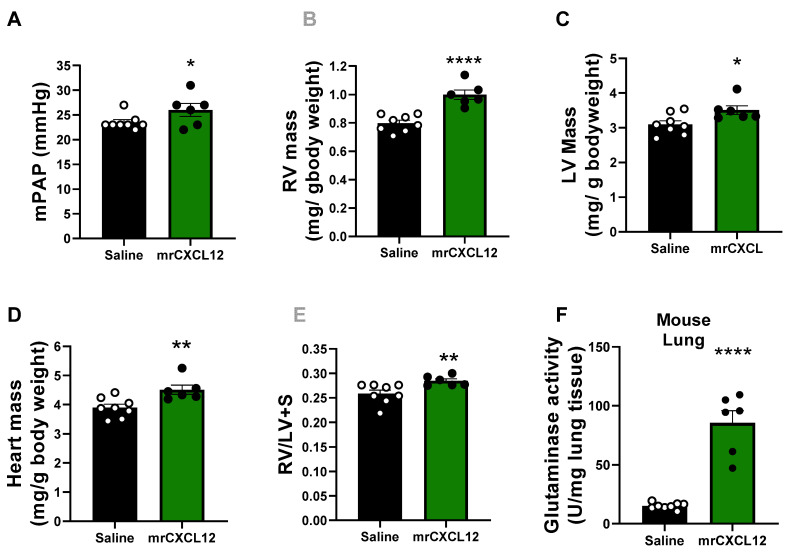

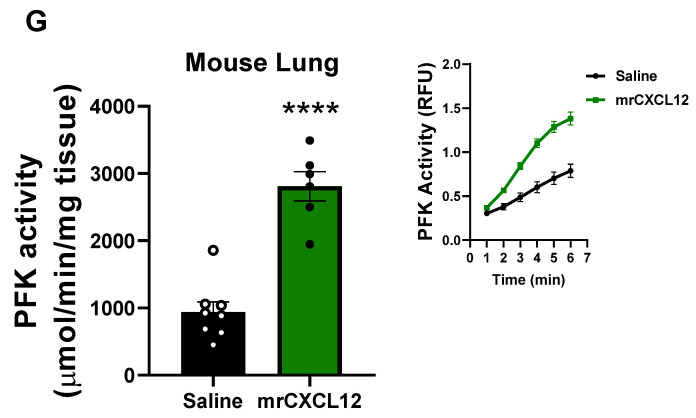
**Subcutaneous CXCL12 infusion induced hallmark characteristics of PAH in female mice.** (**A**) Mice infused with mrCXCL12 or vehicle control for 3 wks were analyzed for pulmonary arterial pressure changes (mPAP) via right heart catheterization. (**B**–**E**) Right and left ventricles were dissected away from the septum. Each were weighed to calculate relative heart chamber and total mass and Fulton Index. (n = 6–8). (**F**,**G**) Activity of (**F**) glutaminase and (**G**) phosphofructokinase (PFK) was quantified in lung lysates (n = 6–8). Results were analyzed by Student t-test. * *p* < 0.05, ** *p* < 0.01, **** *p* < 0.0001.

Given our findings in isolated hPAECs and PAH patient samples, experiments were designed to test whether direct instillation of mrCXCL12 into female mice can induce vascular remodeling reminiscent of PAH. Post-pubescent female mice were chosen as females are more susceptible to develop the disease in early adulthood and during childbearing years. The delivery rate chosen was intended to achieve similar concentrations of CXCL12 to those found in the plasma of human PAH patients. After 3 wks of mrCXCL12 infusion s.c., mean pulmonary artery pressure (mPAP) showed a small but significant (*p* < 0.05) increase (Figure 5A) compared to saline control. CXCL12 administration caused significant increases in RV (*p* < 0.0001), LV (*p* < 0.05), and total heart mass (*p* < 0.01); Fulton index (RV mass/LV + S mass) was minimally yet significantly (*p* < 0.01) elevated (Figure 5B–E). Glutaminase and PFK activity of lung homogenates exhibited ~ 4- and 3-fold increases in activity (Figure 5F and Figure 5G), respectively. Overall, these data illustrate that singular CXCL12 administration can partially recapitulate hallmark indicators of experimental PAH in vivo.

## 4. Discussion

The evidence presented herein reveals a novel pathway by which NOX1 regulates activation of the transcription factor Sp1, in lung endothelial cells subjected to hypoxia (recapitulating PAH phenotype) leading to the increased promotion of CXCL12; and, in turn, proliferation and wound closure. Suppression of NOX1 abolished activation of Sp1, CXCL12 and EC proliferation which was restored by recombinant CXCL12. Moreover, the findings uncover a role for CXCL12 in pro-proliferative glycolytic and glutamine signaling both in vitro and in human lungs. Indeed, human recombinant CXCL12 induced phosphorylation and activation of pro-metabolic, pro-proliferative mTOR; and rapamycin blocked wound closure and proliferation. Infusion of recombinant CXCL12 into mice recapitulated hallmark features of PAH including rises in mPAP, RV hypertrophy and attendant metabolic changes. Our work is the first, to our knowledge, to indicate that CXCL12, promotes such phenotypic changes in ECs pivotal to PAH. Finally, in samples from PAH subjects, detection of NOX1-dependent ROS, activated Sp1 and elevated CXCL12 expression spotlight our studies as clinically significant. 

While ECs & SMCs play a role in pulmonary arterial remodeling in PAH, the current study focuses on mechanisms controlling ECs as seminal in this process. To that point, mechanisms governing EC CXCL12 expression and proliferation, emblematic of remodeling in the pulmonary endothelium have, to our knowledge, not been explored. Our data demonstrate a concentration-dependent increase in hPAEC proliferation in response to CXCL12. Furthermore, indicators of proliferation/migration, wound closure, and EdU incorporation, show that a pathological concentration of CXCL12 induces endothelial proliferation and migration and participates in hypoxia-induced hPAEC proliferation and migration. Interestingly, the impact of CXCL12 had been described to be involved in some forms of cancer as a pro-proliferative and pro-migratory factor [19,20,21]. Broad parallels drawn between cancer and PAH [22] provided apt justification for the study of mechanisms controlling CXCL12 in PAH [16,17,18]. Indeed, prior studies had not revealed factors regulating CXCL12. 

With respect to EC phenotype, our novel findings in vitro illustrate that CXCL12 is pivotal to both proliferation and wound closure a proxy for migration (Figure 3), both of which are characteristic of vascular remodeling occurring in vivo in PAH. Our studies add a new dimension to our previous contentions that NOX1 instigates a remodeling response in hPAECs [8,15]. We provide evidence in the current findings that control of CXCL12 signaling is effected via NOX1 upregulation of transcription factor Sp1. In parallel, CXCL12 (Figure 1) expression in hPAEC lysates (Figure 1C–E) rises in response to hypoxia and is abrogated by NOX1 suppression. To corroborate the effects of NOX1 siRNA by alternate means, we employed selective NOX1 inhibitor NOXA1ds [44]. Importantly, hypoxia-induced proliferation in hPAECs is eradicated by the NOX1 inhibitor and this reduced proliferation was restored by replenishment of hrCXCL12 (Figure 3). One limitation of the current study is that we did not interrogate the effect of CXCL12 in medial smooth muscle. It would indeed be interesting to explore whether EC-specific knockout of NOX1 or CXCL12 can repress the chemokine’s levels across the vascular wall. Those studies await the ongoing generation of NOX1 LoxP mice. 

Proceeding on the premise that NOX1-derived ROS initiates this pathway [8,15], it was intriguing to discover reports suggesting to us that Sp1 is redox-sensitive [25,26,27,45,46]. Moreover, our analyses using Transfac^®^, as well as published studies mapping the CXCL12-promoter in a variety of human, rat and mouse samples, revealed to us that Sp1 could be a transcription factor for CXCL12 [23,24]. Thus, we postulated that NOX1 would drive Sp1 phosphorylation/activation and, in turn, promote the expression of CXCL12. This is, in fact, what our findings support (Figure 2). In alignment with this, hypoxia augmented Sp1 mRNA and protein levels, an effect that was obliterated by NOX1 blockade. Consistent with Sp1 being a transcription factor for CXCL12, hypoxia significantly elevated: (a) transcriptionally active Sp1 (TransAm assay); (b) endogenous Sp1-dependent promoter activity by SpRE and the full CXCL12 promoter sequence (luciferase assay). Further to the point, these were disrupted by NOX1 suppression. Taken together, these data provide robust evidence that NOX1-induced Sp1 binds to the CXCL12 promoter and promotes its transcription in pulmonary endothelia. With respect to phenotype, Sp1 triggers EC proliferation and wound closure (Figure 3) and the clinical significance of these findings is underscored by evidence of total and phospho-Sp1 and transcriptionally active Sp1 in lungs from PAH patients.

Our findings demonstrate that clinically relevant CXCL12 levels promote EC proliferation/wound closure (Figure S2) as well as metabolic changes instrumental to EC hyperplasia (Figure 4). Burgeoning evidence supports that glucose and glutamine metabolism underlie vascular proliferation [47]. Generally speaking, across cell types, glycolysis abets mitochondrial ATP production [34] further propelled by glutamine metabolism and its anaplerotic effects [34]. An energy “failsafe” or supplementation is best well known in cancer and PAH as the Warburg effect. In the current study, we report marked upregulation of EC-glucose metabolism by CXCL12 and provide the first evidence that CXCL12 upregulates EC glutamine metabolism. Indeed, CXCL12 directly promoted EC glucose uptake, as well as increased activity of two rate-limiting metabolic enzymes (PFK and G6PD, associated with glycolysis and pentose phosphate pathway, respectively). Additionally, CXCL12 augmented glutaminase activity, which in turn, was partially responsible for glucose uptake (Figure 4G). Moreover, our results indicate that G6PD and glutaminase participate in CXCL12-induced wound closure and proliferation, respectively, in hPAECs (Figure 4). Finally, in human lung lysates, glutaminase activity was significantly elevated in PAH. In aggregate, these findings indicate that CXCL12 plays a critical role in supporting energy requirements for vascular remodeling in PAH. While elaboration of the precise CXCL12-driven signaling that governs these processes is beyond the scope of this work, preliminary findings suggest that CXCL12 influences downstream mTOR signaling, a pathway well known to exert master regulatory control over various metabolic processes [39,40,41]. Indeed, CXCL12 has been implicated as an activator of mTOR signaling previously [48,49], as well as a driver of glycolytic activation/reprogramming in other cell types [20,50], and may therefore utilize this pathway to control EC metabolism.

Finally, we aimed to interrogate the novel influence of CXCL12 on PAH development and metabolic changes in vivo, and toward that end implanted mice with osmotic minipumps releasing a consistent dose of CXCL12 over 3 wks. Intriguingly, we observed slight but significant telltale increases in mPAP (i.e., RV afterload) and RV hypertrophy in CXCL12-treated mice, which are common indicators of PAH-like phenotypes. Further, we observed marked upregulation of both PFK and G6PD activity in lysates from whole lung isolated from these mice, suggesting an induction of pro-proliferative metabolism (Figure 5). Our work indicates for the first time to our knowledge that singular administration of CXCL12, albeit small, can, in fact, model pathological changes germane to PAH. The findings are consistent with a growing body of evidence showing that blockade of CXCL12 blunts the severity of PAH-like phenotypes in models [18,51]. In our hands, despite the robust increases in metabolic indicators we observed in vivo, the modest rises in mPAP and RV and LV mass might appear to lessen the biological significance and be considered a limitation of our studies. However, even modest changes in pressure (approaching 5 mm Hg) and heart morphometry are biologically significant and, perhaps more importantly, it is impossible to know whether the concentrations of mrCXCL12 arriving at the pulmonary vascular endothelial cell in the mouse in vivo matched those found in human PAH. That is, it is plausible that in our model one might envisage markedly lower concentrations at the endothelial interface than expected as a consequence of pharmacokinetics and the intrinsic stability of exogenously administered CXCL12. Moreover, the likelihood is low that plasma CXCL12 elevations alone account for the entirety of the PAH phenotype. That notwithstanding, the primacy of our in vitro and in vivo findings herein substantiate a role for CXCL12 in driving the PAH phenotype. Whether RV hypertrophy is secondary to changes in the pulmonary vasculature as we posit or more a direct action of CXCL12 on the myocardium is still unknown. From our perspective, there is sufficient rationale for both, and future interrogation of these pathways in both cell types will lend deeper insight into the observed clinical link between CXCL12 and PAH severity.

## 5. Conclusions

**Figure 6:**
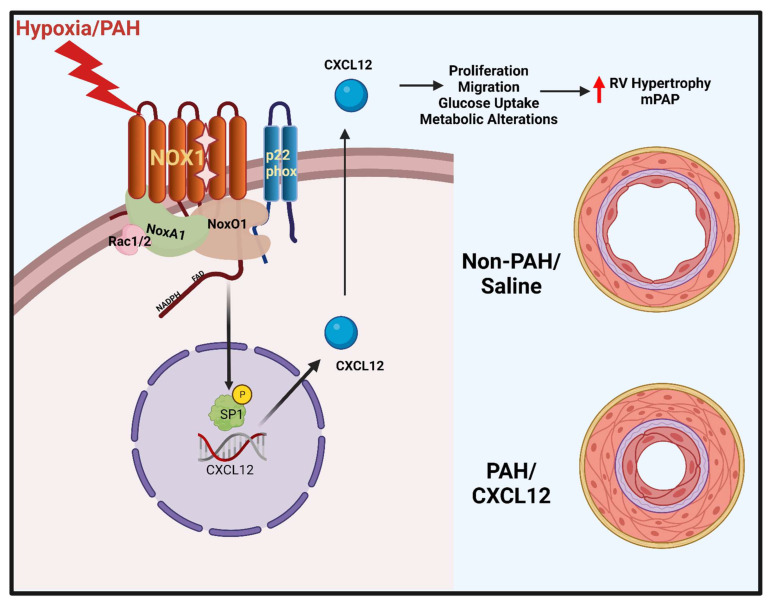
**Working model of NOX1-mediated SP1 regulation of CXCL12 in hypoxia and PAH**. Working model depicting NOX1 (alongside its critical subunits p22*^phox^*, NOXO1, NOXA1, and Rac1/2) induction of CXCL12 via Sp1 following hypoxia. Resultant CXCL12 is postulated to be secreted from cells, leading to downstream promotion of vascular remodeling and PAH symptoms via pro-proliferative metabolic alteration and glucose uptake.

In conclusion, we present a novel ROS-mediated pathway activating Sp1 downstream of NOX1, leading to CXCL12 upregulation. Additionally, we show that this previously unidentified NOX1-Sp1-CXCL12 signaling nexus in pulmonary ECs leads to activation of pro-proliferative metabolism and vascular remodeling in PAH (Figure 6). These data align with our observations in clinical PAH samples, as well as our in vivo model of CXCL12 infusion in mice. Our work further defines a detrimental role for NOX1 signaling in PAH, and identifies Sp1 and CXCL12 as novel downstream mediators in this pathway. In sum, the data inspire the pursuit of these proteins as novel therapeutic targets in a disease with limited treatment options.

## Data Availability

Data is contained within the article and/or Supplementary Material.

## Supplementary Materials

The following are available online at https://www.mdpi.com/article/10.3390/antiox12010071/s1, Figure S1: A. Representative western blot analysis of CXCL12 expression in explanted lung tissue samples from PAH or non-PAH patients (n = 6–10). B. ROS activity in sex and age-matched explanted lung tissue samples from PAH or non-PAH patients. Lysates were treated with scrambled peptide or NoxA1ds and NOX1 activity was assessed using the coumarin boronic acid assay for H2O2. The catalase-inhibitable rates of H2O2 measured as fluorescence (mRFU) compared to controls are reported (n = 6–10). C. ELISA analysis of CXCL12 secretion from hPAEC treated with siCXCL12 as a proof-of-concept for CXCL12 knockdown. D. Quantification of western blot analysis of human lung lysates (blots shown in Figure 2A) for total Sp1/B-actin and p-Sp1/Sp1 (n = 6). E. Western blot of Sp1 expression in cells treated with Scr siRNA or siSp1 as proof of concept for Sp1 knockdown. F. Gating and interpretation of flow cytometry analysis of EdU incorporation. Results were analyzed with and one-way ANOVA and post-hoc Holm-Sidak analysis (A,C), repeated measures ANOVA (B) and Student t-test (D,E). * *p* < 0.05, ** *p* < 0.01, *** *p* < 0.001, **** *p* < 0.0001, Figure S2: A pathological concentration of CXCL12 in human plasma induces human pulmonary endothelial cell proliferation and migration, Figure S3: CXCL12 activates mTOR and rapamycin treatment blocks proliferation and wound closure, Table S1: Human Samples demographics.
