# Supplementary material for: Specificity Protein 1-Mediated Promotion of CXCL12 Advances Endothelial Cell Metabolism and Proliferation in Pulmonary Hypertension
Source: Antioxidants (Basel). 2022 Dec 29;12(1):71. doi: 10.3390/antiox12010071 (PMC9854820; doi:10.3390/antiox12010071)
Supplement: Supplementary file 1 [file antioxidants-12-00071-s001.zip › antioxidants-2056390-supplementary.pdf]

Table S1. Human Samples demographics

Group	Age	Gender	Pathology
Non-PAH	64	M	
	62	M	
	35	M	
	38	M	
	52	F	
	42	F	
	69	F	
	41	F	
	38	F	
	32	F	
PAH	64	M	Idiopathic Pulmonary Fibrosis-associated PH
	59	M	PAH
	52	M	Restrictive Scleroderma ILD and severe PAH
	21	M	Primary PAH
	51	F	Scleroderma-related PAH
	66	F	Scleroderma-related PAH
	37	F	PAH
	50	F	Restrictive Scleroderma and severe PAH
	36	F	Primary PAH
	53	F	Primary PAH

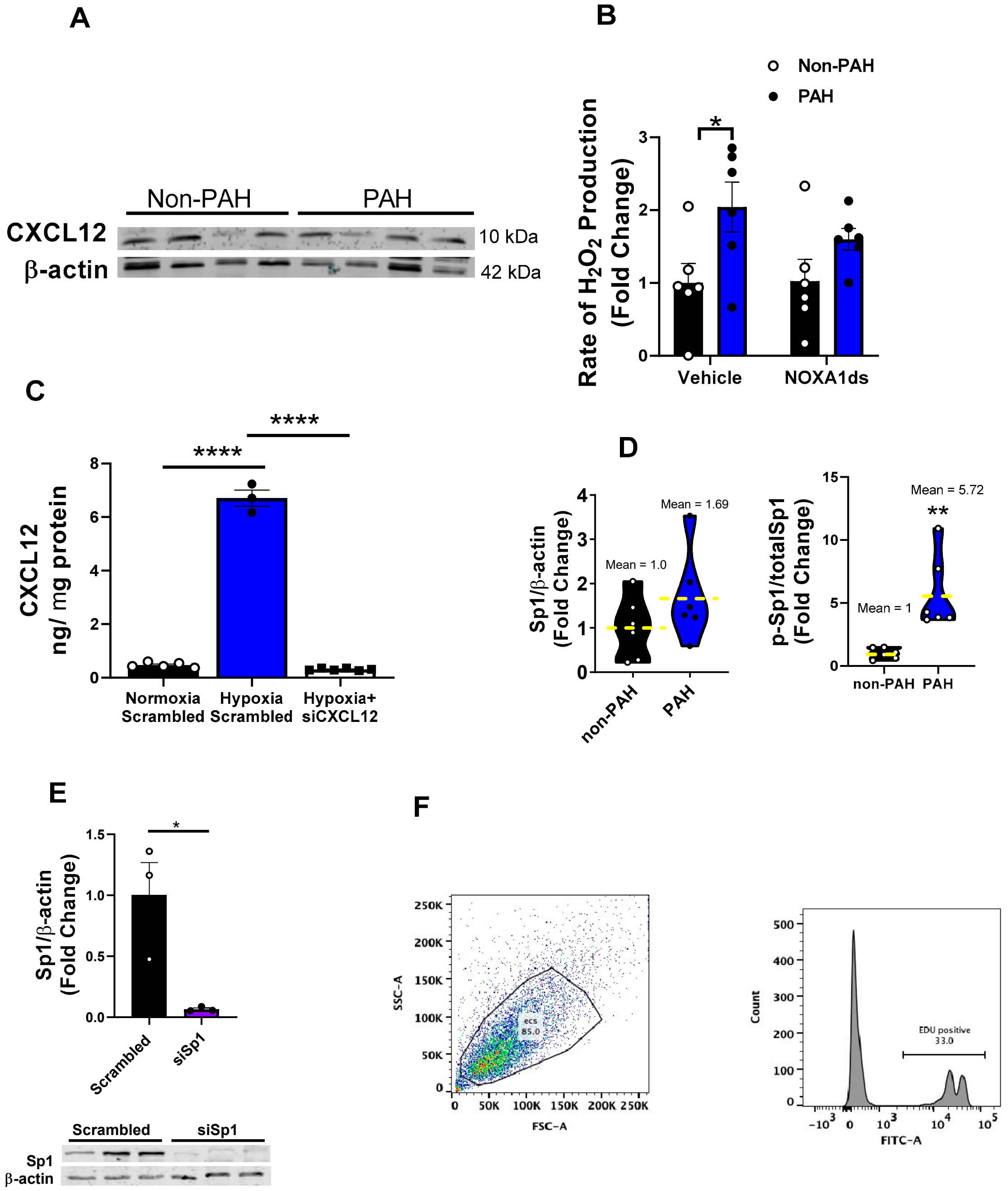

Figure S1. A. Representative western blot analysis of CXCL12 expression in explanted lung tissue samples from PAH or non-PAH patients (n = 6-10). B. ROS activity in sex and age-matched explanted lung tissue samples from PAH or non-PAH patients. Lysates were treated with scrambled peptide or NoxA1ds and NOX1 activity was assessed using the coumarin boronic acid assay for H_2O_2. The catalase-inhibitable rates of H_2O_2 measured as fluorescence (mRFU) compared to controls are reported (n=6-10). C. ELISA analysis of CXCL12 secretion from hPAEC treated with siCXCL12 as a proof-of-concept for CXCL12 knockdown. D. Quantification of western blot analysis of human lung lysates (blots shown in Fig. 2A) for total Sp1/B-actin and p-Sp1/Sp1 (n=6). E. Western blot of Sp1 expression in cells treated with Scr siRNA or siSp1 as proof of concept for Sp1 knockdown. F. Gating and interpretation of flow cytometry analysis of EdU incorporation. Results were analyzed with and one-way ANOVA and post-hoc Holm-Sidak analysis (A, C), repeated measures ANOVA (B) and Student t-test (D,E). * $p<0.05$, ** $p<0.01$, *** $p<0.001$, **** $p<0.0001$

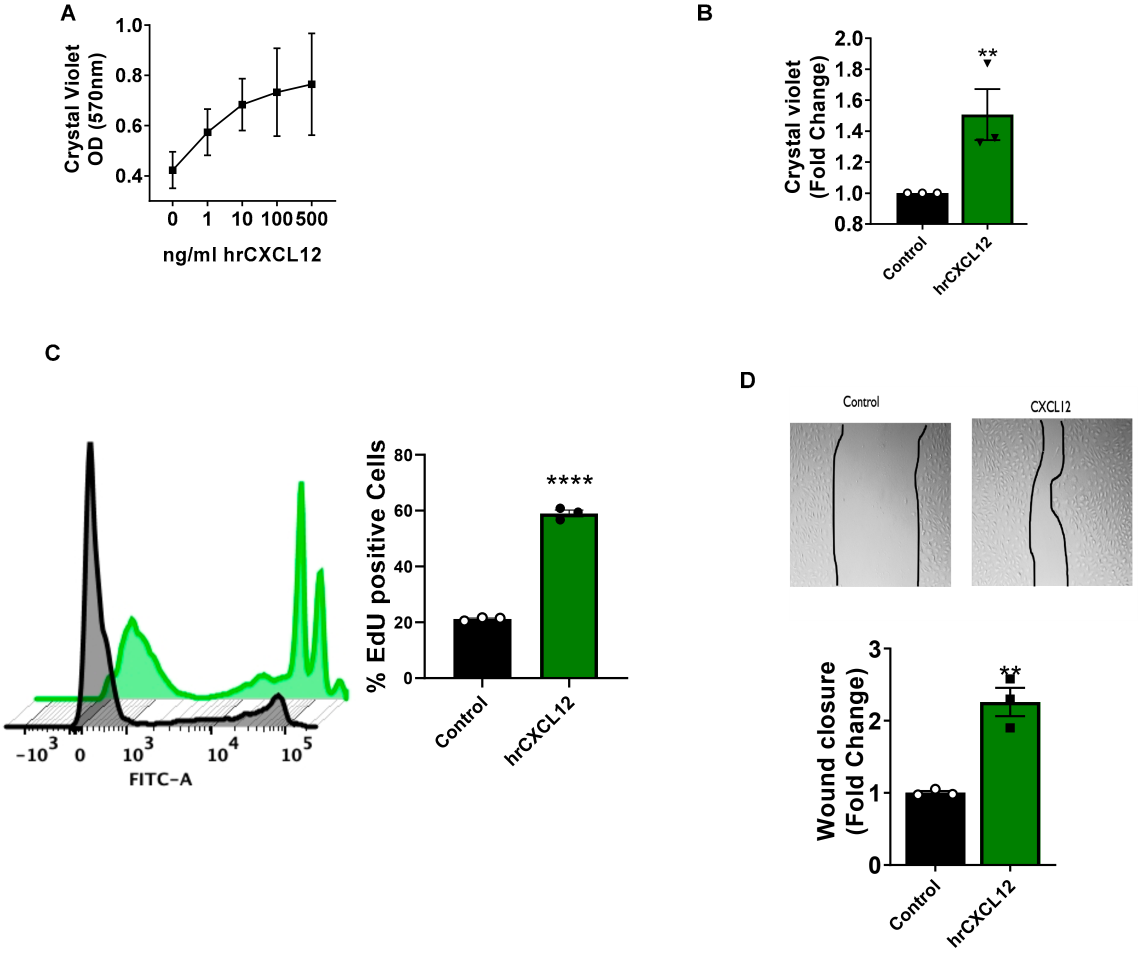

Figure S2. A pathological concentration of CXCL12 in human plasma induces human pulmonary endothelial cell proliferation and migration. A. Concentration-dependent human recombinant CXCL12 (hrCXCL12)-mediated proliferation measured by Crystal Violet in hPAEC at 24 hr. hPAEC proliferation measured by B. Crystal Violet and C. Click-iT™ Edu incorporation following 24 hr treatment with 3 ng/ml hrCXCL12 (n=3-9). D. hPAEC wound closure following 24-hr treatment with hrCXCL12 (n=6) expressed as fold change of treatment vs. vehicle control (PBS) at 24 hr vs. 0 hr. Inset shows images for control- vs. hrCXCL12-treated wounds at 24 hr. Results were analyzed by one-tailed t-test. ** $p<0.01$, **** $p<0.0001$.

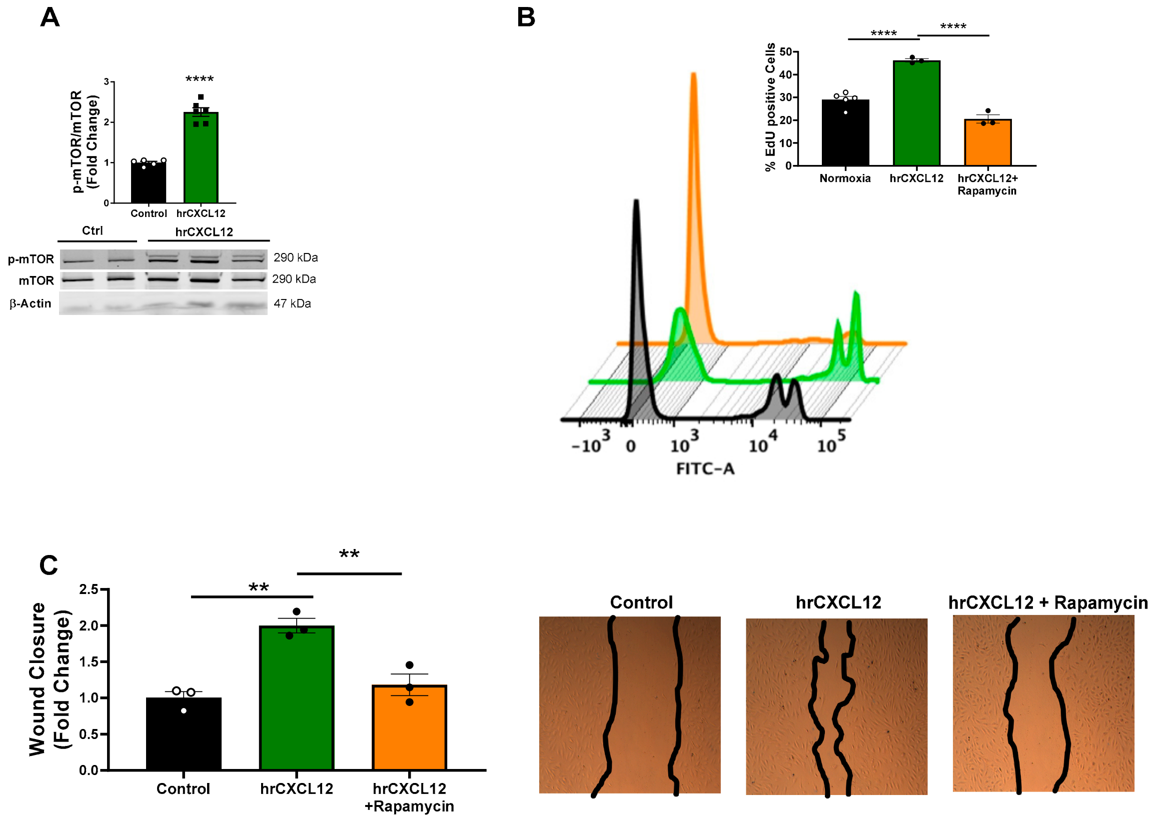

Figure S3. CXCL12 activates mTOR and rapamycin treatment blocks proliferation and wound closure. A. hPAEC were treated with hrCXCL12 or vehicle and probed for p-mTOR and total mTOR. B. Proliferation and C. wound closure measured in hPAEC treated with vehicle or hrCXCL12 or hr CXCL12 for 24hr plus mTOR inhibitor rapamycin (30 min pretreatment). Representative flow cytometry histograms depict populations of EDU positive and negative cells in a sample along the X-axis, with a righthand peak indicating proliferating cells taken as percentage to the total number of cell in the sample. N=3 Results were analyzed by one-tailed t-test (A) and one-way ANOVA with post-hoc Holm-Sidak analysis. (B-C) * $p<0.05$, ** $p<0.01$, *** $p<0.001$, **** $p<0.0001$
